# No evidence for an association of testosterone and cortisol hair concentrations with social decision-making in a large cohort of young adults

**DOI:** 10.1093/scan/nsae090

**Published:** 2024-11-30

**Authors:** Claudia Massaccesi, Lydia Johnson-Ferguson, Josua Zimmermann, Alexander Ehlert, Markus R Baumgartner, Tina M Binz, Denis Ribeaud, Manuel P Eisner, Lilly Shanahan, Heiko Rauhut, Boris B Quednow

**Affiliations:** Department of Cognition, Emotion, and Methods in Psychology, Faculty of Psychology, University of Vienna, Vienna 1010, Austria; Department of Clinical and Health Psychology, Faculty of Psychology, University of Vienna, Vienna 1010, Austria; Experimental and Clinical Pharmacopsychology, Department of Adult Psychiatry and Psychotherapy, University Hospital of Psychiatry Zurich, University of Zurich, Zurich 8032, Switzerland; Jacobs Center for Productive Youth Development, University of Zurich, Zurich 8050, Switzerland; Experimental and Clinical Pharmacopsychology, Department of Adult Psychiatry and Psychotherapy, University Hospital of Psychiatry Zurich, University of Zurich, Zurich 8032, Switzerland; Institute of Sociology, University of Zurich, Zurich 8050, Switzerland; Center for Forensic Hair Analytics, Institute of Forensic Medicine, University of Zurich, Zurich 8057, Switzerland; Center for Forensic Hair Analytics, Institute of Forensic Medicine, University of Zurich, Zurich 8057, Switzerland; Jacobs Center for Productive Youth Development, University of Zurich, Zurich 8050, Switzerland; Jacobs Center for Productive Youth Development, University of Zurich, Zurich 8050, Switzerland; Institute of Criminology, University of Cambridge, Cambridge CB3 9DA, United Kingdom; Jacobs Center for Productive Youth Development, University of Zurich, Zurich 8050, Switzerland; Department of Psychology, University of Zurich, Zurich 8050, Switzerland; Institute of Sociology, University of Zurich, Zurich 8050, Switzerland; Experimental and Clinical Pharmacopsychology, Department of Adult Psychiatry and Psychotherapy, University Hospital of Psychiatry Zurich, University of Zurich, Zurich 8032, Switzerland; Jacobs Center for Productive Youth Development, University of Zurich, Zurich 8050, Switzerland; Neuroscience Center Zurich, ETH Zurich and University of Zurich, Zurich 8057, Switzerland

**Keywords:** social decision-making, prosocial behavior, testosterone, cortisol, hair hormones

## Abstract

Prior research has established that testosterone is an important modulator of social decision-making. However, evidence on the relationship between basal testosterone levels, commonly measured in saliva or blood, and social behavior has been inconsistent due to methodological shortcomings. Additionally, it has been suggested that cortisol might moderate the association between basal testosterone and social behavior. The present study examined how individual differences in cumulative hair testosterone map onto social decision-making under consideration of a potential modulating role of hair cortisol in a large community sample of young adults (*N* = 1002). We observed a negative association between hair testosterone and trust behavior (odds ratio = 0.84) and a positive association with self-reported aggressive behavior (*β* = 0.08). The effects were small and became nonsignificant after controlling for key covariates of steroid hormones in hair (e.g. hair color, contraceptives, and use of psychoactive substances). Hair testosterone levels were not significantly associated with any other social behavior examined, and no modulating effects of hair cortisol were found. Overall, these findings provide no evidence for a role of basal testosterone hair concentrations in human social decision-making and do not indicate that hair cortisol moderates hair testosterone’s effects on social behavior.

## Introduction

Every day, individuals make prosocial decisions to incur personal costs, such as sharing or donating money, to benefit familiar and unfamiliar others. These prosocial behaviors are fundamental for societal well-being and are influenced by a complex interplay of biological, situational, and social factors (Post 2005, Schroeder and Graziano 2015).

Previous research indicates a pivotal role of hormones, including testosterone, in shaping social behavior. Importantly, testosterone has been shown to influence social decision-making in both antisocial (competitive) and prosocial (cooperative) directions. Influential theoretical models, such as the challenge hypothesis (Wingfield et al. 1990) and the biosocial model of status (Mazur 1985), propose that endogenous testosterone facilitates aggressive and dominant behaviors when they are beneficial for reproduction or for increasing/maintaining status. While studies in nonhuman animals observed a direct relationship between endogenous testosterone and aggressive or dominant behavior (Nelson and Trainor 2007), this association has been shown to be relatively weak in humans (*r* = 0.08, Archer et al. 2005; *r* = 0.06, Geniole et al. 2020).

Importantly, in humans, high social status is achieved and maintained not only through aggressive, antisocial behavior but also via prosociality. Particularly in contexts free of social threats, testosterone may promote socially desirable behaviors, like cooperation and fairness, as such behaviors increase status by building reputation and positive social image (Eisenegger et al. 2011, Terburg and van Honk 2013). Using economic games adapted from game theory and testosterone administration, prior research provided evidence supporting this account. For example, exogenous testosterone administration increased, rather than reduced, fair bargaining in the ultimatum game (Eisenegger et al. 2010). Similarly, exogenous testosterone administration reduced trust (socially threatening context) but increased reciprocity (nonthreatening context) in the trust game (Boksem et al. 2013). Testosterone administration also promoted punishment of unfair offers (which may be perceived as a challenge to one’s status) and reward of high offers (generosity) in the ultimatum game (Dreher et al. 2016).

While research suggests that acute changes in testosterone, such as those induced by exogenous administration, may be more relevant to changes in human social behavior (Carré and Olmstead 2015, Knight et al. 2020), some studies have also indicated associations between basal testosterone concentrations and social decision-making. Two studies found that individuals with high salivary baseline testosterone show higher fairness preferences, being more likely to reject unfair offers in the ultimatum game than those with low testosterone (Burnham 2007, Mehta and Beer 2010). Higher testosterone was also associated with higher dominant behavior among high-rank individuals and higher prosocial behavior among lower-rank ones (Inoue et al. 2017).

The Dual Hormone Hypothesis posits that the association between basal testosterone and social behavior is moderated by cortisol, specifically that the positive association between basal testosterone and status-relevant behavior is more robust when cortisol levels are low and weaker when cortisol levels are high (Mehta and Josephs 2010, Mehta and Prasad 2015). However, recent meta-analyses revealed only a weak testosterone × cortisol interaction effect on measures relating to status-seeking, antisocial, and prosocial behavior, with some studies providing supporting findings while others showing opposite or null effects (Dekkers et al. 2019, Grebe et al. 2019, Knight et al. 2020).

Overall, while some of the existent evidence suggests a role of basal testosterone in promoting prosocial and antisocial behaviors depending on the social context (i.e. presence of a threat to status), prior research presents several limitations that hinder conclusions on the relationship between basal testosterone and social decision-making. First, most previous studies included small sample sizes and focused exclusively on males or females, ignoring the possible moderator effect of sex and preventing the generalizability of findings. In addition, most studies included a single economic game and, thus, focused on a specific aspect of prosocial behavior, limiting a broader understanding of basal testosterone’s effects on social decision-making. Crucially, basal levels of testosterone have been primarily determined via saliva or blood samples collected at a single time point. However, endogenous levels of testosterone and other hormones, such as cortisol, fluctuate during the day and dynamically change due to a wide range of environmental and contextual factors (e.g. diurnal fluctuations, Matchock et al. 2007). Basal hormone assessment via single saliva/blood samples is potentially confounded by these fluctuations in hormone secretions. In contrast, hair analysis represents a reliable, noninvasive tool for assessing basal hormonal concentrations. Hair hormonal levels reflect cumulative exposure over several months and are thus less affected by circadian or acute stress-related variations (Stalder and Kirschbaum 2012, Binz et al. 2018, Voegel et al. 2020). Despite its numerous advantages, to date, only a few studies have employed hair analysis to examine the relationship between basal testosterone and behavior. Hair testosterone was recently shown to predict prosocial conformity (Duell et al. 2021), inhibitory control (Shields et al. 2019), and externalizing behaviors (Grotzinger et al. 2018) in adolescents, as well as risk-taking in adult men (Ronay et al. 2018). Consistently with the Dual Hormone Hypothesis, most of these effects of hair testosterone were observed only at low levels of hair cortisol. Nevertheless, to date, studies examining the relationship between cumulative hair testosterone levels and social decision-making are lacking.

The present study aimed to fill this knowledge gap and overcome previous research limitations in examining the association between basal testosterone and social decision-making. To this aim, we assessed 3-month cumulative testosterone concentrations via hair analysis and social decision-making using a series of socioeconomic games (trust, ultimatum, dictator, and public good games) in a large community sample of young adults (*N* = 1002). In line with the social status hypothesis, we hypothesized that testosterone levels would affect game behavior depending on whether a threat to the social status is present. Specifically, we expected a negative relationship between hair testosterone and prosocial behavior in contexts characterized by social threat (i.e. decision to trust in the trust game, Boksem et al. 2013; minimal acceptance offer in the ultimatum game, Prasad et al. 2017; contribution in the public goods game) and a positive relationship between hair testosterone and prosocial behavior in contexts free of social threat (offer in the ultimatum, Eisenegger et al. 2010; i.e. reciprocity in the trust game, Boksem et al. 2013). Concerning the dictator game, we expected higher testosterone to be associated with unfair offers (proactive dominance; Prasad et al. 2019). Drawing on the Dual Hormone Hypothesis, we additionally measured hair cortisol to test for possible interacting effects of testosterone and cortisol on prosocial and antisocial behavior.

## Materials and methods

### Sample

Data were collected as part of the “The Zurich Project on the Social Development from Childhood to Adulthood” (z-proso; Ribeaud et al. 2022). The present study includes 1002 (503 female) participants, aged between 19 and 22 years (*M* = 20.57, SD = 0.38), who provided hair samples in Wave 8 in 2018. The study was conducted in accordance with the Declaration of Helsinki (World Medical Association 2013) and approved by the local research ethics committees (Kantonale Ethikkommission Zürich for hair data collection and Ethics Committee of the Faculty of Social Sciences and Humanities of the University of Zurich for decision-making games and survey data). Participants provided written informed consent. Hair sampling and decision-making games were carried out at the Decision Science Lab at the ETH Zurich.

### Social decision-making tasks

**Figure 1.**
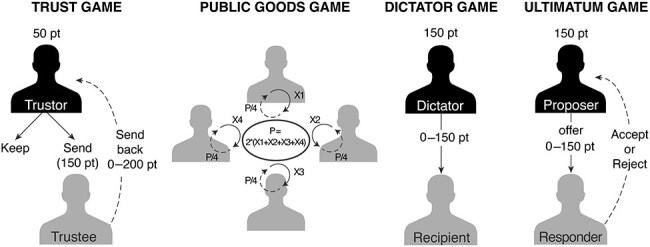
Schematic representations of the social decision-making games. X = amount contributed to the public good; P = payoff.

Following the z-proso main study, participants performed a series of one-shot social decision-making games in the following order: the trust game (Berg et al. 1995) assessing trust and reciprocity, the public goods game (Ledyard 1995) assessing cooperation, the dictator game (Bolton et al. 1998) assessing proactive dominance, and last the ultimatum game (Güth et al. 1982) assessing fairness and reactive dominance (Fig. 1). Participants performed the games separated by cubicles and instructed not to communicate with each other. All games were played using the strategy vector method (Selten 1967) to ensure anonymity. At the end of the study, the points earned in one randomly selected game were paid out (10 points = 1 CHF; average payout = 7.9 CHF).

In the trust game, participants played once as trustor and once as trustee. The trustor and trustee received an initial endowment of 50 points. The trustor had to choose whether to keep the 50 points or send them to the trustee (decision to trust). In the latter case, the points were tripled by the experimenter and the trustee received 150 points. If the trustor decided to send the 50 points, the trustee could transfer any amount between 0 and 200 points back to the trustor (amount returned). This amount was transferred back to the trustor without multiplication. Trustors were also asked to guess how many points (from 0 to 200) they would expect back if they had trusted the trustee (belief about other’s trustworthiness). In the four-player public goods game, participants simultaneously decided how much to contribute of their initial endowment of 50 points into a joint project (public good). The total contributions were then multiplied by a factor of 2 and evenly distributed among all participants, regardless of their individual contributions. In the dictator game, participants (in the role of dictators) received an endowment of 150 points and decided how to divide it between themselves and the other anonymous player (recipient). In the ultimatum game, participants played once as proposer and once as responder. The proposer received an endowment of 150 points and had to make an offer on how to split the points between themselves and the responder (amount offered). The responder was asked to indicate the minimal amount at which they would still accept the offer (minimum acceptable offer, MAO). If the offer was below the MAO, it was rejected and both players received 0 points. Otherwise, both players received their stake.

### Questionnaires

The “Aggression” and “Prosociality” subscales of the Social Behavior Questionnaire (SBQ; Tremblay et al. 1991, Murray et al. 2017) were used to assess participants’ aggressive behavior, including physical, indirect, instrumental/dominant, and reactive aggression, and prosocial behavior, including empathy and helping. Items are rated on a 5-point Likert scale from “Never” to “Very Often”.

### Hair analysis

Hair samples were collected from the scalp and segmented into 3 cm reflecting the past 3 months prior sampling. Body hair was collected when scalp hair was insufficiently long (8.7%). Hair samples were collected on the same testing session in which participants performed the decision-making games. Testosterone and cortisol concentrations were determined using liquid chromatography–tandem mass spectrometry (for a detailed description, see Scholz et al. 2022).

### Statistical analysis

Hair cortisol and testosterone concentrations were log-transformed due to right-skewness. Based on the observed sex differences in testosterone [*t*(636.5) = 11.04, *P* < .001; *M*  = 2.01 (males); *M* = 0.49 (females)] but not in cortisol [*t*(889.22) = −0.53, *P* = .599; *M* = 5.38 (males); *M* = 5.60 (females)], testosterone was standardized (*z*-score) within sex, while cortisol was standardized (*z*-score) across sex (Mehta and Josephs 2010, Mehta et al. 2015b; as in Grotzinger et al. 2018). Thus, high scores on the testosterone distribution reflect high levels relative to other individuals of the same sex.

We conducted hierarchical multiple regression analysis for each outcome variable in two steps (logistic regression for binary outcome variables and linear regression otherwise). In Step 1, the main effects of testosterone and cortisol were entered as predictors. In Step 2, the testosterone × cortisol interaction was added (Mehta et al. 2015a). Both steps included sex as a covariate. We then also examined if sex moderated the effects of hormones on the dependent variables by adding to the models the interaction term between sex and the other predictors (testosterone and cortisol). All regression models were additionally fitted with the inclusion of additional covariates related to hair analysis and/or social decision-making: body mass index (BMI), hormonal contraception (estrogen–progestin and progestin/none), hair color (blond and brown/black), hair type (scalp and body), period of the year of hair collection (collection calendar week), and hair concentration of psychoactive substances (opioids, stimulants, cannabis, and MDMA, all log-transformed) (Stalder and Kirschbaum 2012, Parrott and Downey 2017, Binz et al. 2018, Quednow 2020, Carrillo Vázquez et al. 2022, Voegel et al. 2022, Johnson-Ferguson et al. 2023, Ehlert et al. 2024). All confidence intervals were bootstrapped. We reported unstandardized (*b*) and standardized (*β*) regression coefficients. Complete results tables of the regression models can be found in the Supplementary material. Analyses were conducted in R (version: 4.3.0; R Core Team, 2023).

## Results

### Trust game

**Figure 2.**
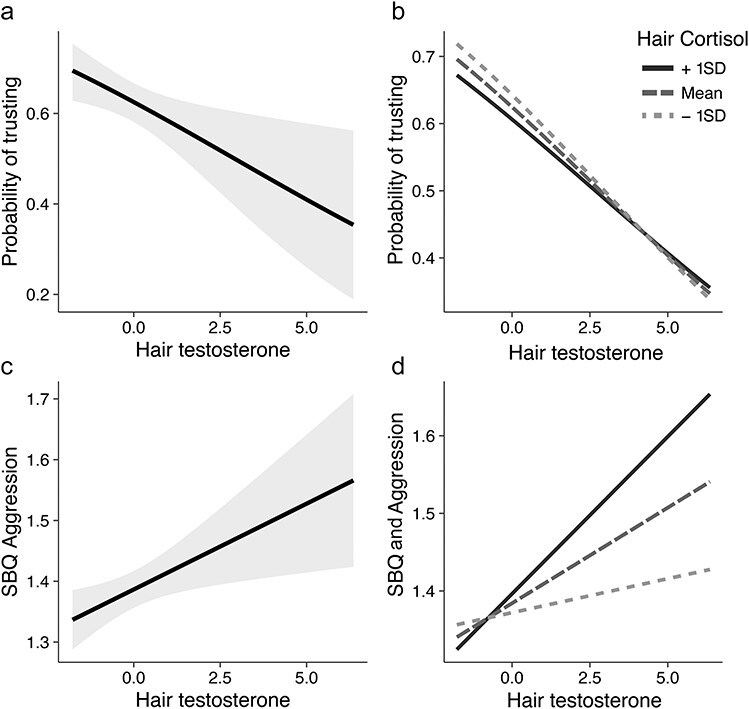
Predicted probability to trust in the trust game (a, b) and self-reported aggressive behavior (SBQ) (c, d) as a function of hair testosterone, independent of hair cortisol (a–c) and at mean, +1SD, and − 1SD hair cortisol levels (b–d). Hair testosterone and cortisol levels are log-transformed and standardized.

Hair testosterone significantly predicted participants’ decision to trust (i.e. to keep or send the endowment to the other player). Specifically, higher concentrations of hair testosterone were associated with a lower probability of trusting the other player [odds ratio = 0.84, 95% confidence interval (CI) [0.73, 0.96], *z*(995) = −2.62, *P* = .009; see Table 1 and Fig. 2a]. However, after including the covariates (BMI, hormonal contraception, hair color, hair type, period of the year of hair collection, and hair concentration of psychoactive substances), this association became nonsignificant (*P* = .112, Supplementary Table S2). No significant main or interaction effects of cortisol were observed (all *P* > .25; see Table 1 and Fig. 2b). The regression models on the belief about other’s trustworthiness (testosterone: *β* = −0.01, *b* = −0.53, 95% CI[−3.27, 2.21]; cortisol: *β* = −0.02, *b* = −1.03, 95% CI[−3.67, 1.72]; testosterone × cortisol: *β* = 0.04, *b* = 1.66, 95% CI[−0.79, 4.07]) and on the amount returned (positive reciprocity; testosterone: *β* = 0.01, *b* = 0.25, 95% CI[−2.29, 2.67]; cortisol: *β* = −0.04, *b* = −1.67, 95% CI −4.27, 0.95; testosterone × cortisol: *β* = 0.02, *b* = 0.90, 95% CI[−1.53, 3.19]) did not reveal a significant main or interaction effect of testosterone and cortisol (all *t* < 1.27, all *P* > .20, Supplementary Tables S4–S6). We did not observe any significant two- (sex × testosterone/cortisol) or three-way (sex × testosterone × cortisol) interactions (all *P* > .15, Supplementary Tables S1, S3, and S5).

**Table 1.** Results from logistic regression analyses predicting decision to trust as a function of testosterone and cortisol concentrations.

	Odds Ratio	Std. error	*z*	*P*
**Step 1**				
Intercept	1.67	0.09	5.51	<.001
Testosterone	0.84	0.07	−2.62	.009
Cortisol	0.92	0.07	−1.15	.250
Sex	1.24	0.13	1.59	.112
Model fit	*χ* ^2^(3) = 12.40, *P* = .01			
*R* ^2^ Tjur	0.013			
**Step 2**				
Intercept	1.66	0.09	5.45	<.001
Testosterone	0.84	0.07	−2.63	.009
Cortisol	0.93	0.07	−1.15	.251
Sex	1.24	0.13	1.58	.114
Testosterone × cortisol	1.02	0.06	0.30	.765
Model fit	*χ* ^2^(4) = 12.49, *P* = .01			
*R* ^2^ Tjur	0.013			

### Public goods game

No significant main or interaction effects of hair testosterone and cortisol were observed on cooperation, assessed as the amount of money the participants contributed to the public pot (testosterone: *β* = −0.03, *b* = −0.43, 95% CI[−1.43, 0.67]; cortisol: *β* = −0.03, *b* = −0.52, 95% CI [−1.60, 0.52]; testosterone × cortisol: *β* = 0.02, *b* = 0.38, 95% CI[−0.57, 1.38]) (all *t* < −0.97, all *P* > .33). The inclusion of covariates did not alter the results pattern (Supplementary Table S14). When examining the moderating effect of sex moderated the effects on cooperation, we did not observe any significant two- (sex × testosterone/cortisol) or three-way (sex × testosterone × cortisol) interactions (all *P* > .55, Supplementary Table S13).

### Dictator game

No significant main or interaction effects of hair testosterone and cortisol were observed on proactive dominance, assessed as the amount of money shared with the other player in the dictator game (testosterone: *β* = −0.01, *b* = −0.25, 95% CI[−2.43, 1.69]; cortisol: *β* = 0.01, *b* = 0.39, 95% CI[−1.70, 2.46]; testosterone × cortisol: *β* = −0.01, *b* = −0.44, 95% CI[−2.32, 1.24]) (all *t* < 0.48, all *P* > .63). The inclusion of covariates did not alter the results pattern (Supplementary Table S8). When examining the moderating effect of sex moderated the effects on the amount of money shared, we did not observe any significant two- (sex × testosterone/cortisol) or three-way (sex × testosterone × cortisol) interactions (all *P* > .27, Supplementary Table S7).

### Ultimatum Game

No significant main or interaction effects of hair testosterone and cortisol were observed on fairness (amount offered, testosterone: *β* = 0.01, *b* = 0.37, 95% CI[−1.50, 2.09]; cortisol: *β* = −0.05, *b* = −1.21, 95% CI[−2.95, 0.43]; testosterone × cortisol: *β* = −0.01, *b* = −0.34, 95% CI[−1.97, 1.32]) or on the level of MAO (reactive dominance; testosterone: *β* = −0.01, *b* = 0.25, 95% CI[−2.06, 1.48]; cortisol: *β* = 0.05, *b* = 1.34, 95% CI[−0.53, 3.30]; testosterone × cortisol: *β* = 0.01, *b* = 0.27, 95% CI [−1.26, 1.79]) at the ultimatum game (all *t* < 1.53, all *P* > .13, Supplementary Tables S9–S12). Including covariates or the sex interaction term in the models did not alter the results pattern (Supplementary Tables S9 and S11).

### Self-reported aggression

Hair testosterone significantly predicted the reported frequency of aggressive behavior (*β* = 0.08, *b* = 0.03, 95% CI[0.01, 0.05]; *t*(997) = 2.54, *P* = .011; see Table 2 and Fig. 2c). Specifically, participants with higher levels of hair testosterone scored higher at the SBQ aggression subscale. However, after including the covariates (BMI, hormonal contraception, hair color, hair type, period of the year of hair collection, and hair concentration of psychoactive substances), this association only showed a trend to significance (*P* = .071, Supplementary Table S16). No significant main or interaction effects of cortisol were observed (all *t* < 1.33, all *P* > .18; see Table 2 and Fig. 2d). When examining the moderating effect of sex moderated the effects on self-reported aggression, we did not observe any significant two- (sex × testosterone/cortisol) or three-way (sex × testosterone × cortisol) interactions (all *P* > .08, Supplementary Table S15).

**Table 2.** Results from linear regression analyses predicting aggressive behavior (SBQ aggression) as a function of testosterone and cortisol concentrations.

	*b*	*β*	Std. error	*t*	*P*
**Step 1**					
Intercept	1.39	−0.09	0.02	89.99	<.001
Testosterone	0.03	0.08	0.01	2.54	.011
Cortisol	0.01	0.03	0.01	1.02	.309
Sex	0.06	0.19	0.02	2.97	.003
Model fit	*F*(3997) = 5.75, *P* < 0.001				
*R* ^2^/*R*^2^ adjusted	0.017/0.014				
**Step 2**					
Intercept	1.38	−0.10	0.02	89.47	<.001
Testosterone	0.02	0.07	0.01	2.18	.030
Cortisol	0.01	0.03	0.01	1.09	.276
Sex	0.06	0.18	0.02	2.92	.004
Testosterone × cortisol	0.02	0.05	0.01	1.56	.119
Model fit	*F*(4996) = 4.92, *P* < .001				
*R* ^2^/*R*^2^ adjusted	0.019/0.015				

### Self-reported prosociality

No significant main or interaction effects of hair cortisol and testosterone were observed on reported frequency of prosocial behavior (SBQ prosociality; testosterone: *β* = 0.04, *b* = 0.02, 95% CI[−0.01, 0.05]; cortisol: *β* = 0.01, *b* = 0.01, 95% CI[−0.03, 0.04]; testosterone × cortisol: *β* = −0.05, *b* = −0.03, 95% CI[−0.05, 0.01]) (all *t* < −1.62, all *P* > .11, Supplementary Table S18). When examining the moderating effect of sex on self-reported aggression, we did not observe any significant two- (sex × testosterone/cortisol) or three-way (sex × testosterone × cortisol) interactions (all *P* > .08, Supplementary Table S17).

## Discussion

The present study extends prior research on basal testosterone and social behavior by investigating their relationship in a large community sample of young adults using a range of social decision-making games and cumulative hormone concentrations measured in hair. We observed a small effect of hair testosterone on trust behavior and self-reported aggressive behavior, which was not statistically significant after controlling for other factors known to affect hair hormone concentrations, such as hair color, contraception, and use of psychoactive substances (Carrillo Vázquez et al. 2022, Johnson-Ferguson et al. 2023, Ehlert et al. 2024). Hair testosterone levels were not significantly associated with any other social behavior examined, nor with self-reported prosociality, and no significant modulating effects of hair cortisol were found.

We observed a negative relationship between hair testosterone and trust behavior, independent of sex. Higher hair testosterone levels predicted a lower probability of trusting the other player by transferring them the initial endowment. The effect of basal testosterone was specific to trust behavior, as no significant modulation of trust reciprocity was observed. The direction of the finding aligns with previous evidence from administration studies, which showed that exogenous testosterone administration reduces trust (Bos et al. 2010, 2012, Boksem et al. 2013). However, the negative association had a small effect size (odds ratio = 0.84) and was not significant (*P* = .112) after controlling for various confounders of hair hormone measurement.

Similar to previous findings in adolescents (Grotzinger et al. 2018), basal hair testosterone was positively associated with self-reported aggressive behavior. Again, the effect size was small (*β* = 0.08), as would be expected considering the results of previous meta-analyses (e.g. Archer et al. 2005, Geniole et al. 2020), and was not significant (*P* = .071) after the inclusion of various covariates that could affect hair hormone measurement. In contrast with the Dual Hormone Hypothesis and with the results from Grotzinger et al. (2018), we did not observe any moderating role of cortisol in the testosterone–aggression relationship.

Importantly, we found no evidence for a relationship between basal hair testosterone and the other examined social behaviors, that is fairness, proactive and reactive dominance, and cooperation. Considering the large sample size, these null findings indicate that cumulative testosterone levels —measured in hair— are not linked to social decision-making.

Of note, cumulative hair testosterone can be considered a more “trait-like” component of testosterone (Grotzinger et al. 2018). Despite its advantage of being less prone to fluctuations due to external/internal factors (time of the day, mood, etc.), the present study suggests that hair testosterone is not a more robust predictor of social behavior. Acute changes in testosterone concentration in response to social stimuli and situations may be more relevant than cumulative measures in explaining social behavior. Future studies should aim to compare testosterone’s basal and dynamic responses, for example, by collecting hair samples together with blood samples at different time points (e.g. before, during, and after completion of socioeconomic games) in large, gender-balanced samples (Geniole and Carré 2018, Knight et al. 2020).

Our findings also highlight the importance of accounting for variables affecting hormone concentrations in hair, such as sex, hair color, and use of contraceptives in females, in future research (Stalder and Kirschbaum 2012, Parrott and Downey 2017, Binz et al. 2018, Quednow 2020, Carrillo Vázquez et al. 2022, Voegel et al. 2022, Johnson-Ferguson et al. 2023). Despite their known effect on hair hormone concentrations, these key covariates are rarely considered when examining the association between hair hormones and behavior.

Despite the efforts to overcome previous research limitations (low statistical power, gender/sex imbalance, unstable validity of salivary methods, etc.; Knight et al. 2020), we did not observe a modulatory effect of cortisol, providing no support to the Dual Hormone Hypothesis (Mehta and Josephs 2010). Two previous studies showed a positive relationship between hair testosterone concentrations and social behavior (aggression and peer conformity) in adolescents only at low hair cortisol levels (Grotzinger et al. 2018, Duell et al. 2021). Nevertheless, evidence in support of the Dual Hormone Hypothesis has been largely inconsistent across studies (Dekkers et al. 2019, Grebe et al. 2019, Knight et al. 2020). It is possible that dynamic, rather than basal, cortisol changes, such as those related to acute stress exposure, represent a stronger moderator of the association between testosterone and status-seeking behavior. Accordingly, a rise in cortisol levels following acute stress suppressed the association between testosterone and retaliation (Prasad et al. 2017), empathic accuracy (Nitschke and Bartz 2020), and resource allocation (Prasad et al. 2019).

It is important to consider some limitations of the present study. First, although we included a large, sex-balanced sample, the generalizability of the results is limited due to the narrowed age range considered (19–22 years). However, the high homogeneity in age can also be seen as an advantage, as the absence of strong age effects might have enabled the detection of small correlations between hormones and behavior. Future research should nevertheless extend the investigation to younger and older individuals and examine the impact of testosterone developmental trajectories on social behavior across the lifespan. Furthermore, the study did not include any direct measure of social status or status-seeking behavior (Knight et al. 2020). Future work should replicate these results using more direct measures of social status. Nevertheless, the employed social decision-making games are effective tools for assessing prosocial and antisocial behavior, and some of the measures collected can be interpreted in the context of status-seeking strategies (see, e.g., Boksem et al. 2013, Prasad et al. 2017, 2019). Last, the study included only one-shot games, neglecting potential effects on repeated interactions, which involves, for example, learning processes. Thus, future research should examine the relationship between hormones and repeated social interactions via repeated games.

In conclusion, we showed in a large sample of young adults that endogenous cumulative testosterone assessed via hair analysis does not significantly predict social decision-making, especially when accounting for key covariates of steroid hormones in hair. We also did not observe an interaction effect of basal hair cortisol levels, providing no support to the Dual Hormone Hypothesis. Considering the observed lack of association between testosterone and a broad range of examined social behaviors, our data indicate that basal hair testosterone (and cortisol) levels do not significantly modulate human social decision-making in young adults.

## Supplementary Material

nsae090_Supp

## Data Availability

The data underlying this article will be shared on reasonable request to the corresponding author.
