# Correction to: No evidence for an association of testosterone and cortisol hair concentrations with social decision-making in a large cohort of young adults

**DOI:** 10.1093/scan/nsaf002

**Published:** 2025-01-21

This is a correction to: Claudia Massaccesi, Lydia Johnson-Ferguson, Josua Zimmermann, Alexander Ehlert, Markus R Baumgartner, Tina M Binz, Denis Ribeaud, Manuel P Eisner, Lilly Shanahan, Heiko Rauhut, Boris B Quednow, No evidence for an association of testosterone and cortisol hair concentrations with social decision-making in a large cohort of young adults, *Social Cognitive and Affective Neuroscience*, Volume 19, Issue 1, 2024, nsae090, https://doi.org/10.1093/scan/nsae090.

The originally published version of this manuscript contained an error in the spelling of author Heiko Rauhut’s name. This error has been corrected.

