# Supplementary material for: No evidence for an association of testosterone and cortisol hair concentrations with social decision-making in a large cohort of young adults
Source: Soc Cogn Affect Neurosci. 2024 Nov 30;19(1):nsae090. doi: 10.1093/scan/nsae090 (PMC11642610; doi:10.1093/scan/nsae090)
Supplement: nsae090_Supp [file nsae090_supp.zip › nsae090_Supp/scan-24-199-File002.docx]

**Supplementary Material**

**No evidence for an association of testosterone and cortisol hair concentrations with social decision-making in a large cohort of young adults**

Claudia Massaccesi*, Lydia Johnson-Ferguson, Josua Zimmermann,
Alexander Ehlert, Markus R. Baumgartner, Tina M. Binz, Denis Ribeaud, Manuel P. Eisner, Lilly Shanahan°, Heiko Rahut°, Boris B. Quednow°

°Equal contribution

*Correspondence: Claudia Massaccesi, claudia.massaccesi@univie.ac.at

Table of Contents

[1. Trust game: decision to trust 3](#_Toc168071245)

[Table S1. 3](#_Toc168071246)

[Table S2. 4](#_Toc168071247)

[2. Trust game: belief about other’s trustworthiness 5](#_Toc168071248)

[Table S3. 5](#_Toc168071249)

[Table S4. 6](#_Toc168071250)

[3. Trust game: amount returned 7](#_Toc168071251)

[Table S5. 7](#_Toc168071252)

[Table S6. 8](#_Toc168071253)

[4. Dictator game: amount shared 9](#_Toc168071254)

[Table S7. 9](#_Toc168071255)

[Table S8. 10](#_Toc168071256)

[5. Ultimatum game: amount offered 11](#_Toc168071257)

[Table S9. 11](#_Toc168071258)

[Table S10. 12](#_Toc168071259)

[6. Ultimatum game: minimum acceptable offer 14](#_Toc168071260)

[Table S11. 14](#_Toc168071261)

[Table S12. 15](#_Toc168071262)

[7. Public goods game: amount contributed 16](#_Toc168071263)

[Table S13. 16](#_Toc168071264)

[Table S14. 17](#_Toc168071265)

[8. Self-reported aggression 18](#_Toc168071266)

[Table S15. 18](#_Toc168071267)

[Table S16. 19](#_Toc168071268)

[9. Self-reported prosociality 20](#_Toc168071269)

[Table S17. 20](#_Toc168071270)

[Table S18. 21](#_Toc168071271)

In the following tables, we report the complete results of the regression models for each dependent variable. CI = 95% confidence interval (not bootstrapped).

# Trust game: decision to trust

**Table S1.** Results from logistic regression analyses of “decision to trust” in the trust game including the interaction term with sex.

|  | **Decision to trust** | | | | |
| --- | --- | --- | --- | --- | --- |
| **Step 1** | *Odds Ratios* | *std. Beta* | *CI* | *standardized CI* | *p* |
| (Intercept) | 1.67 | 1.67 | 1.39 – 2.00 | 1.39 – 2.01 | **<0.001** |
| Testosterone | 0.77 | 0.77 | 0.64 – 0.93 | 0.64 – 0.93 | **0.006** |
| Cortisol | 0.96 | 0.96 | 0.80 – 1.16 | 0.80 – 1.16 | 0.672 |
| Sex | 1.23 | 1.23 | 0.95 – 1.60 | 0.95 – 1.60 | 0.120 |
| Testosterone × Sex | 1.19 | 1.19 | 0.92 – 1.56 | 0.92 – 1.56 | 0.189 |
| Cortisol × Sex | 0.92 | 0.92 | 0.70 – 1.20 | 0.71 – 1.20 | 0.541 |
| Observations | 995 | | | | |
| R^2^ Tjur | 0.015 | | | | |
| χ²(7) = 15.90, p = 0.03 | | | | | |

| **Step 2** | *Odds Ratios* | *std. Beta* | *CI* | *standardized CI* | *p* |
| --- | --- | --- | --- | --- | --- |
| (Intercept) | 1.68 | 1.68 | 1.40 – 2.03 | 1.40 – 2.03 | **<0.001** |
| Testosterone | 0.78 | 0.78 | 0.64 – 0.94 | 0.64 – 0.94 | **0.011** |
| Cortisol | 0.96 | 0.96 | 0.80 – 1.16 | 0.80 – 1.16 | 0.674 |
| Sex | 1.20 | 1.20 | 0.92 – 1.56 | 0.92 – 1.57 | 0.181 |
| Testosterone × Cortisol | 0.94 | 0.94 | 0.78 – 1.12 | 0.78 – 1.12 | 0.491 |
| Testosterone × Sex | 1.15 | 1.15 | 0.88 – 1.52 | 0.88 – 1.52 | 0.301 |
| Cortisol × Sex | 0.93 | 0.93 | 0.71 – 1.21 | 0.71 – 1.21 | 0.571 |
| Testosterone × Cortisol × Sex | 1.17 | 1.17 | 0.91 – 1.50 | 0.92 – 1.50 | 0.220 |
| Observations | 995 | | | | |
| R^2^ Tjur | 0.016 | | | | |
| χ²(7) = 15.90, p = 0.03 | | | | | |

**Table S2.** Results from logistic regression analyses of “decision to trust” in the trust game including covariates.

|  | **Decision to trust** | | | | |
| --- | --- | --- | --- | --- | --- |
| **Step 1** | *Odds Ratios* | *std. Beta* | *CI* | *standardized CI* | *p* |
| (Intercept) | 4.94 | 1.70 | 2.01 – 12.25 | 1.23 – 2.38 | **0.001** |
| Testosterone | 0.89 | 0.89 | 0.78 – 1.03 | 0.78 – 1.03 | 0.112 |
| Cortisol | 0.95 | 0.95 | 0.83 – 1.09 | 0.83 – 1.09 | 0.446 |
| Sex | 1.61 | 1.61 | 1.18 – 2.21 | 1.18 – 2.21 | **0.003** |
| CollectionWeek | 0.98 | 0.89 | 0.96 – 1.00 | 0.78 – 1.02 | 0.082 |
| Hair type | 0.73 | 0.73 | 0.45 – 1.21 | 0.45 – 1.21 | 0.220 |
| Contraceptives | 1.64 | 1.64 | 1.09 – 2.49 | 1.09 – 2.49 | **0.018** |
| BMI | 0.97 | 0.89 | 0.94 – 1.00 | 0.78 – 1.02 | 0.092 |
| Hair color | 0.78 | 0.78 | 0.55 – 1.09 | 0.55 – 1.09 | 0.144 |
| Opioids | 0.92 | 0.92 | 0.81 – 1.05 | 0.80 – 1.05 | 0.195 |
| Cannabis | 1.01 | 1.01 | 0.92 – 1.11 | 0.87 – 1.18 | 0.876 |
| Stimulants | 0.96 | 0.99 | 0.54 – 1.81 | 0.85 – 1.17 | 0.900 |
| MDMA | 1.03 | 1.04 | 0.93 – 1.14 | 0.88 – 1.24 | 0.632 |
| Observations | 990 | | | | |
| R^2^ Tjur | 0.032 | | | | |
| χ²(12) = 32.10, p = 0.00 | | | | | |

| **Step2** | *Odds Ratios* | *std. Beta* | *CI* | *standardized CI* | *p* |
| --- | --- | --- | --- | --- | --- |
| (Intercept) | 4.94 | 1.70 | 2.01 – 12.24 | 1.22 – 2.38 | **0.001** |
| Testosterone | 0.89 | 0.89 | 0.77 – 1.03 | 0.77 – 1.03 | 0.110 |
| Cortisol | 0.95 | 0.95 | 0.83 – 1.09 | 0.83 – 1.09 | 0.445 |
| Sex | 1.61 | 1.61 | 1.18 – 2.21 | 1.18 – 2.21 | **0.003** |
| CollectionWeek | 0.98 | 0.89 | 0.96 – 1.00 | 0.78 – 1.02 | 0.082 |
| CollectionWeek | 0.73 | 0.73 | 0.45 – 1.21 | 0.45 – 1.21 | 0.216 |
| Hair type | 1.64 | 1.64 | 1.09 – 2.49 | 1.09 – 2.49 | **0.019** |
| Contraceptives | 0.97 | 0.89 | 0.94 – 1.00 | 0.78 – 1.02 | 0.091 |
| BMI | 0.78 | 0.78 | 0.55 – 1.09 | 0.55 – 1.09 | 0.148 |
| Hair color | 0.92 | 0.92 | 0.81 – 1.05 | 0.80 – 1.05 | 0.202 |
| Opioids | 1.01 | 1.01 | 0.92 – 1.11 | 0.87 – 1.18 | 0.879 |
| Cannabis | 0.96 | 0.99 | 0.54 – 1.81 | 0.85 – 1.17 | 0.900 |
| Stimulants | 1.03 | 1.04 | 0.93 – 1.14 | 0.88 – 1.24 | 0.627 |
| Testosterone × Cortisol | 1.01 | 1.01 | 0.90 – 1.15 | 0.90 – 1.15 | 0.837 |
| Observations | 990 | | | | |
| R^2^ Tjur | 0.032 | | | | |
| χ²(13) = 32.14, p = 0.00 | | | | | |

# Trust game: belief about other’s trustworthiness

**Table S3.** Results from logistic regression analyses of “belief about other’s trustworthiness” in the trust game including the interaction term with sex.

|  | **Belief about other’s trustworthiness** | | | | |
| --- | --- | --- | --- | --- | --- |
| **Step 1** | *Estimates* | *std. Beta* | *CI* | *standardized CI* | *p* |
| (Intercept) | 58.89 | -0.06 | 55.07 – 62.70 | -0.14 – 0.03 | **<0.001** |
| Testosterone | -1.75 | -0.04 | -5.60 – 2.10 | -0.13 – 0.05 | 0.372 |
| Cortisol | -0.61 | -0.01 | -4.52 – 3.31 | -0.10 – 0.08 | 0.762 |
| Sex | 4.77 | 0.11 | -0.64 – 10.18 | -0.01 – 0.23 | 0.084 |
| Testosterone × Sex | 2.49 | 0.06 | -3.01 – 7.99 | -0.07 – 0.18 | 0.374 |
| Cortisol × Sex | -0.93 | -0.02 | -6.44 – 4.58 | -0.15 – 0.11 | 0.741 |
| Observations | 993 | | | | |
| R^2^ / R^2^ adjusted | 0.005 / -0.000 | | | | |
| F(5,987) = 0.96, p = 0.44 | | | | | |

| **Step 2** | *Estimates* | *std. Beta* | *CI* | *standardized CI* | *p* |
| --- | --- | --- | --- | --- | --- |
| (Intercept) | 58.88 | -0.06 | 55.03 – 62.74 | -0.14 – 0.03 | **<0.001** |
| Testosterone | -1.76 | -0.04 | -5.70 – 2.18 | -0.13 – 0.05 | 0.381 |
| Cortisol | -0.60 | -0.01 | -4.52 – 3.31 | -0.10 – 0.08 | 0.762 |
| Sex | 4.07 | 0.09 | -1.41 – 9.56 | -0.03 – 0.22 | 0.145 |
| Testosterone × Cortisol | 0.04 | 0.00 | -3.62 – 3.69 | -0.08 – 0.09 | 0.984 |
| Testosterone × Sex | 1.86 | 0.04 | -3.75 – 7.46 | -0.09 – 0.17 | 0.516 |
| Cortisol × Sex | -0.68 | -0.02 | -6.20 – 4.84 | -0.14 – 0.11 | 0.809 |
| Testosterone × Cortisol × Sex | 3.12 | 0.07 | -1.93 – 8.17 | -0.04 – 0.19 | 0.226 |
| Observations | 993 | | | | |
| R^2^ / R^2^ adjusted | 0.008 / 0.001 | | | | |
| F(7,985) = 1.14, p = 0.34 | | | | | |

**Table S4.** Results from logistic regression analyses of “belief about other’s trustworthiness” in the trust game including covariates.

|  | **Belief about other’s trustworthiness** | | | | |
| --- | --- | --- | --- | --- | --- |
| **Step 1** | *Estimates* | *std. Beta* | *CI* | *standardized CI* | *p* |
| (Intercept) | 70.35 | -0.21 | 51.94 – 88.77 | -0.37 – -0.06 | **<0.001** |
| Testosterone | -0.00 | -0.00 | -2.85 – 2.84 | -0.07 – 0.07 | 0.999 |
| Cortisol | -0.67 | -0.02 | -3.50 – 2.16 | -0.08 – 0.05 | 0.643 |
| Sex | 10.21 | 0.24 | 3.81 – 16.61 | 0.09 – 0.38 | **0.002** |
| CollectionWeek | -0.21 | -0.03 | -0.69 – 0.26 | -0.09 – 0.04 | 0.378 |
| Hair type | -4.47 | -0.10 | -14.77 – 5.83 | -0.34 – 0.13 | 0.395 |
| Contraceptives | 10.92 | 0.25 | 2.73 – 19.12 | 0.06 – 0.44 | **0.009** |
| BMI | -0.57 | -0.06 | -1.22 – 0.09 | -0.12 – 0.01 | 0.091 |
| Hair color | 3.54 | 0.08 | -3.09 – 10.16 | -0.07 – 0.23 | 0.295 |
| Opioids | -2.26 | -0.05 | -4.93 – 0.41 | -0.12 – 0.01 | 0.097 |
| Cannabis | -0.54 | -0.02 | -2.40 – 1.33 | -0.09 – 0.05 | 0.573 |
| Stimulants | -5.05 | -0.03 | -16.92 – 6.82 | -0.11 – 0.04 | 0.404 |
| MDMA | 0.16 | 0.01 | -1.95 – 2.26 | -0.07 – 0.08 | 0.884 |
| Observations | 988 | | | | |
| R^2^ / R^2^ adjusted | 0.022 / 0.010 | | | | |
| F(12,975) = 1.84, p = 0.04 | | | | | |

| **Step 2** | *Estimates* | *std. Beta* | *CI* | *standardized CI* | *p* |
| --- | --- | --- | --- | --- | --- |
| (Intercept) | 70.31 | -0.22 | 51.91 – 88.72 | -0.38 – -0.07 | **<0.001** |
| Testosterone | -0.40 | -0.01 | -3.31 – 2.51 | -0.08 – 0.06 | 0.787 |
| Cortisol | -0.59 | -0.01 | -3.43 – 2.24 | -0.08 – 0.05 | 0.682 |
| Sex | 10.11 | 0.23 | 3.71 – 16.50 | 0.09 – 0.38 | **0.002** |
| CollectionWeek | -0.22 | -0.03 | -0.70 – 0.26 | -0.09 – 0.03 | 0.368 |
| Hair type | -4.82 | -0.11 | -15.13 – 5.49 | -0.35 – 0.13 | 0.359 |
| Contraceptives | 10.85 | 0.25 | 2.65 – 19.04 | 0.06 – 0.44 | **0.010** |
| BMI | -0.58 | -0.06 | -1.24 – 0.08 | -0.12 – 0.01 | 0.083 |
| Hair color | 3.81 | 0.09 | -2.83 – 10.45 | -0.07 – 0.24 | 0.260 |
| Opioids | -2.13 | -0.05 | -4.80 – 0.55 | -0.12 – 0.01 | 0.119 |
| Cannabis | -0.57 | -0.02 | -2.43 – 1.30 | -0.09 – 0.05 | 0.551 |
| Stimulants | -5.03 | -0.03 | -16.90 – 6.84 | -0.11 – 0.04 | 0.406 |
| MDMA | 0.20 | 0.01 | -1.90 – 2.31 | -0.07 – 0.08 | 0.850 |
| Testosterone × Cortisol | 1.68 | 0.04 | -0.85 – 4.21 | -0.02 – 0.10 | 0.192 |
| Observations | 988 | | | | |
| R^2^ / R^2^ adjusted | 0.024/0.011 | | | | |
| F(13,974) = 1.83, p = 0.03 | | | | | |

# Trust game: amount returned

**Table S5.** Results from logistic regression analyses of “amount returned” in the trust game including the interaction term with sex.

|  | **Amount returned** | | | | |
| --- | --- | --- | --- | --- | --- |
| **Step 1** | *Estimates* | *std. Beta* | *CI* | *standardized CI* | *p* |
| (Intercept) | 79.68 | -0.03 | 76.12 – 83.24 | -0.12 – 0.06 | **<0.001** |
| Testosterone | -0.81 | -0.02 | -4.44 – 2.83 | -0.11 – 0.07 | 0.664 |
| Cortisol | -1.49 | -0.04 | -5.15 – 2.16 | -0.13 – 0.05 | 0.423 |
| Sex | 2.62 | 0.06 | -2.43 – 7.66 | -0.06 – 0.19 | 0.310 |
| Testosterone × Sex | 2.13 | 0.05 | -3.03 – 7.30 | -0.07 – 0.18 | 0.418 |
| Cortisol × Sex | -0.43 | -0.01 | -5.58 – 4.72 | -0.14 – 0.12 | 0.871 |
| Observations | 992 | | | | |
| R^2^ / R^2^ adjusted | 0.004 / -0.002 | | | | |
| F(5,986) = 0.69, p = 0.63 | | | | | |

| **Step 2** | *Estimates* | *std. Beta* | *CI* | *standardized CI* | *p* |
| --- | --- | --- | --- | --- | --- |
| (Intercept) | 79.29 | -0.04 | 75.70 – 82.88 | -0.13 – 0.05 | **<0.001** |
| Testosterone | -1.36 | -0.03 | -5.06 – 2.34 | -0.13 – 0.06 | 0.471 |
| Cortisol | -1.40 | -0.04 | -5.06 – 2.26 | -0.13 – 0.06 | 0.452 |
| Sex | 3.17 | 0.08 | -1.95 – 8.29 | -0.05 – 0.20 | 0.225 |
| Testosterone × Cortisol | 2.78 | 0.07 | -0.71 – 6.27 | -0.02 – 0.15 | 0.118 |
| Testosterone × Sex | 2.84 | 0.07 | -2.42 – 8.09 | -0.06 – 0.20 | 0.289 |
| Cortisol × Sex | -0.58 | -0.01 | -5.74 – 4.58 | -0.14 – 0.11 | 0.826 |
| Testosterone × Cortisol × Sex | -3.51 | -0.09 | -8.29 – 1.26 | -0.20 – 0.03 | 0.149 |
| Observations | 992 | | | | |
| R^2^ / R^2^ adjusted | 0.006 / -0.001 | | | | |
| F(7,984) = 0.87, p = 0.53 | | | | | |

**Table S6.** Results from logistic regression analyses of “amount returned” in the trust game including covariates.

|  | **Amount returned** | | | | |
| --- | --- | --- | --- | --- | --- |
| **Step 1** | *Estimates* | *std. Beta* | *CI* | *standardized CI* | *p* |
| (Intercept) | 95.69 | -0.05 | 78.50 – 112.87 | -0.20 – 0.10 | **<0.001** |
| Testosterone | 1.18 | 0.03 | -1.49 – 3.85 | -0.04 – 0.09 | 0.388 |
| Cortisol | -1.24 | -0.03 | -3.89 – 1.40 | -0.10 – 0.03 | 0.356 |
| Sex | 7.07 | 0.18 | 1.11 – 13.04 | 0.03 – 0.32 | **0.020** |
| CollectionWeek | -0.06 | -0.01 | -0.51 – 0.38 | -0.07 – 0.05 | 0.776 |
| Hair type | -5.60 | -0.14 | -15.27 – 4.06 | -0.38 – 0.10 | 0.255 |
| Contraceptives | 5.89 | 0.15 | -1.75 – 13.53 | -0.04 – 0.34 | 0.131 |
| BMI | -0.54 | -0.06 | -1.15 – 0.07 | -0.12 – 0.01 | 0.084 |
| Hair color | -2.58 | -0.06 | -8.76 – 3.60 | -0.22 – 0.09 | 0.412 |
| Opioids | -2.43 | -0.06 | -4.95 – 0.09 | -0.13 – 0.00 | 0.058 |
| Cannabis | -1.47 | -0.06 | -3.21 – 0.27 | -0.13 – 0.01 | 0.098 |
| Stimulants | 7.48 | 0.05 | -3.60 – 18.56 | -0.02 – 0.12 | 0.186 |
| MDMA | -0.33 | -0.01 | -2.30 – 1.64 | -0.09 – 0.06 | 0.742 |
| Observations | 987 | | | | |
| R^2^ / R^2^ adjusted | 0.021 / 0.009 | | | | |
| F(12,974) = 1.72, p = 0.06 | | | | | |

| **Step 2** | *Estimates* | *std. Beta* | *CI* | *standardized CI* | *p* |
| --- | --- | --- | --- | --- | --- |
| (Intercept) | 95.66 | -0.05 | 78.48 – 112.85 | -0.21 – 0.10 | **<0.001** |
| Testosterone | 1.01 | 0.02 | -1.71 – 3.74 | -0.04 – 0.09 | 0.465 |
| Cortisol | -1.21 | -0.03 | -3.85 – 1.44 | -0.10 – 0.04 | 0.371 |
| Sex | 7.02 | 0.17 | 1.05 – 13.00 | 0.03 – 0.32 | **0.021** |
| CollectionWeek | -0.07 | -0.01 | -0.51 – 0.38 | -0.07 – 0.05 | 0.770 |
| Hair type | -5.77 | -0.14 | -15.45 – 3.92 | -0.38 – 0.10 | 0.243 |
| Contraceptives | 5.85 | 0.15 | -1.79 – 13.50 | -0.04 – 0.33 | 0.133 |
| BMI | -0.55 | -0.06 | -1.16 – 0.07 | -0.12 – 0.01 | 0.081 |
| Hair color | -2.46 | -0.06 | -8.66 – 3.73 | -0.21 – 0.09 | 0.435 |
| Opioids | -2.37 | -0.06 | -4.90 – 0.15 | -0.13 – 0.00 | 0.066 |
| Cannabis | -1.48 | -0.06 | -3.22 – 0.26 | -0.13 – 0.01 | 0.095 |
| Stimulants | 7.49 | 0.05 | -3.60 – 18.57 | -0.02 – 0.12 | 0.185 |
| MDMA | -0.31 | -0.01 | -2.28 – 1.66 | -0.09 – 0.07 | 0.757 |
| Testosterone × Cortisol | 0.74 | 0.02 | -1.65 – 3.12 | -0.04 – 0.08 | 0.544 |
| Observations | 987 | | | | |
| R^2^ / R^2^ adjusted | 0.021 / 0.008 | | | | |
| F(13,973) = 1.61, p = 0.08 | | | | | |

# Dictator game: amount shared

**Table S7.** Results from logistic regression analyses of “amount shared” in the dictator game including the interaction term with sex.

|  | **Amount shared** | | | | |
| --- | --- | --- | --- | --- | --- |
| **Step 1** | *Estimates* | *std. Beta* | *CI* | *standardized CI* | *p* |
| (Intercept) | 66.36 | 0.04 | 63.63 – 69.09 | -0.04 – 0.13 | **<0.001** |
| Testosterone | 0.80 | 0.03 | -1.95 – 3.55 | -0.06 – 0.11 | 0.567 |
| Cortisol | -0.53 | -0.02 | -3.33 – 2.27 | -0.11 – 0.07 | 0.711 |
| Sex | -2.63 | -0.09 | -6.50 – 1.24 | -0.21 – 0.04 | 0.183 |
| Testosterone × Sex | -2.21 | -0.07 | -6.14 – 1.72 | -0.20 – 0.06 | 0.270 |
| Cortisol × Sex | 1.90 | 0.06 | -2.04 – 5.85 | -0.07 – 0.19 | 0.344 |
| Observations | 990 | | | | |
| R^2^ / R^2^ adjusted | 0.004 / -0.001 | | | | |
| F(5,984) = 0.77, p = 0.57 | | | | | |

| **Step 2** | *Estimates* | *std. Beta* | *CI* | *standardized CI* | *p* |
| --- | --- | --- | --- | --- | --- |
| (Intercept) | 66.31 | 0.04 | 63.56 – 69.07 | -0.05 – 0.13 | **<0.001** |
| Testosterone | 0.74 | 0.02 | -2.08 – 3.56 | -0.07 – 0.12 | 0.607 |
| Cortisol | -0.52 | -0.02 | -3.32 – 2.28 | -0.11 – 0.07 | 0.715 |
| Sex | -2.35 | -0.08 | -6.28 – 1.58 | -0.20 – 0.05 | 0.242 |
| Testosterone × Cortisol | 0.27 | 0.01 | -2.35 – 2.89 | -0.08 – 0.09 | 0.839 |
| Testosterone × Sex | -1.92 | -0.06 | -5.94 – 2.09 | -0.19 – 0.07 | 0.347 |
| Cortisol × Sex | 1.81 | 0.06 | -2.14 – 5.76 | -0.07 – 0.19 | 0.368 |
| Testosterone × Cortisol × Sex | -1.35 | -0.04 | -4.96 – 2.27 | -0.16 – 0.07 | 0.464 |
| Observations | 990 | | | | |
| R^2^ / R^2^ adjusted | 0.005 / -0.002 | | | | |
| F(7,982) = 0.65, p = 0.71 | | | | | |

**Table S8.** Results from logistic regression analyses of “amount shared” in the dictator game including covariates.

|  | **Amount shared** | | | | |
| --- | --- | --- | --- | --- | --- |
| **Step 1** | *Estimates* | *std. Beta* | *CI* | *standardized CI* | *p* |
| (Intercept) | 62.19 | 0.12 | 48.99 – 75.40 | -0.04 – 0.27 | **<0.001** |
| Testosterone | -0.11 | -0.00 | -2.15 – 1.93 | -0.07 – 0.06 | 0.917 |
| Cortisol | -0.15 | -0.00 | -2.18 – 1.88 | -0.07 – 0.06 | 0.887 |
| Sex | -4.05 | -0.13 | -8.63 – 0.54 | -0.28 – 0.02 | 0.083 |
| CollectionWeek | 0.32 | 0.06 | -0.02 – 0.66 | -0.00 – 0.12 | 0.066 |
| Hair type | -0.57 | -0.02 | -7.99 – 6.86 | -0.26 – 0.22 | 0.881 |
| Contraceptives | -1.20 | -0.04 | -7.09 – 4.70 | -0.23 – 0.15 | 0.690 |
| BMI | 0.07 | 0.01 | -0.40 – 0.54 | -0.05 – 0.07 | 0.765 |
| Hair color | -1.81 | -0.06 | -6.57 – 2.94 | -0.21 – 0.09 | 0.454 |
| Opioids | -0.05 | -0.00 | -1.98 – 1.88 | -0.07 – 0.06 | 0.959 |
| Cannabis | 1.01 | 0.05 | -0.33 – 2.35 | -0.02 – 0.12 | 0.140 |
| Stimulants | 12.31 | 0.11 | 3.80 – 20.82 | 0.03 – 0.18 | **0.005** |
| MDMA | -1.93 | -0.10 | -3.45 – -0.42 | -0.18 – -0.02 | **0.012** |
| Observations | 985 | | | | |
| R^2^ / R^2^ adjusted | 0.018 / 0.006 | | | | |
| F(12,972) = 1.47, p = 0.13 | | | | | |

| **Step 2** | *Estimates* | *std. Beta* | *CI* | *standardized CI* | *p* |
| --- | --- | --- | --- | --- | --- |
| (Intercept) | 62.21 | 0.12 | 49.00 – 75.42 | -0.03 – 0.28 | **<0.001** |
| Testosterone | 0.02 | 0.00 | -2.06 – 2.10 | -0.07 – 0.07 | 0.985 |
| Cortisol | -0.17 | -0.01 | -2.21 – 1.86 | -0.07 – 0.06 | 0.868 |
| Sex | -4.02 | -0.13 | -8.60 – 0.57 | -0.28 – 0.02 | 0.086 |
| CollectionWeek | 0.32 | 0.06 | -0.02 – 0.66 | -0.00 – 0.12 | 0.065 |
| Hair type | -0.45 | -0.01 | -7.89 – 6.99 | -0.25 – 0.23 | 0.906 |
| Contraceptives | -1.17 | -0.04 | -7.07 – 4.72 | -0.23 – 0.15 | 0.697 |
| BMI | 0.08 | 0.01 | -0.39 – 0.55 | -0.05 – 0.07 | 0.750 |
| Hair color | -1.90 | -0.06 | -6.67 – 2.86 | -0.22 – 0.09 | 0.433 |
| Opioids | -0.09 | -0.00 | -2.04 – 1.85 | -0.07 – 0.06 | 0.924 |
| Cannabis | 1.02 | 0.05 | -0.32 – 2.36 | -0.02 – 0.12 | 0.136 |
| Stimulants | 12.31 | 0.11 | 3.79 – 20.82 | 0.03 – 0.18 | **0.005** |
| MDMA | -1.95 | -0.10 | -3.46 – -0.43 | -0.18 – -0.02 | **0.012** |
| Testosterone × Cortisol | -0.54 | -0.02 | -2.36 – 1.27 | -0.08 – 0.04 | 0.557 |
| Observations | 985 | | | | |
| R^2^ / R^2^ adjusted | 0.018 / 0.005 | | | | |
| F(13,971) = 1.38, p = 0.16 | | | | | |

# Ultimatum game: amount offered

**Table S9.** Results from logistic regression analyses of “amount offered” in the ultimatum game including the interaction term with sex.

|  | **Amount offered** | | | | |
| --- | --- | --- | --- | --- | --- |
| **Step 1** | *Estimates* | *std. Beta* | *CI* | *standardized CI* | *p* |
| (Intercept) | 74.35 | 0.01 | 72.12 – 76.58 | -0.08 – 0.09 | **<0.001** |
| Testosterone | 0.03 | 0.00 | -2.21 – 2.28 | -0.09 – 0.09 | 0.977 |
| Cortisol | -3.53 | -0.14 | -5.81 – -1.25 | -0.23 – -0.05 | **0.002** |
| Sex | -0.02 | -0.00 | -3.17 – 3.14 | -0.13 – 0.12 | 0.992 |
| Testosterone × Sex | 0.52 | 0.02 | -2.69 – 3.73 | -0.11 – 0.15 | 0.751 |
| Cortisol × Sex | 4.56 | 0.18 | 1.34 – 7.78 | 0.05 – 0.31 | **0.006** |
| Observations | 990 | | | | |
| R^2^ / R^2^ adjusted | 0.011 / 0.006 | | | | |
| F(5,984) = 2.13, p = 0.06 | | | | | |

| **Step 2** | *Estimates* | *std. Beta* | *CI* | *standardized CI* | *p* |
| --- | --- | --- | --- | --- | --- |
| (Intercept) | 74.36 | 0.01 | 72.10 – 76.61 | -0.08 – 0.10 | **<0.001** |
| Testosterone | 0.04 | 0.00 | -2.27 – 2.34 | -0.09 – 0.09 | 0.975 |
| Cortisol | -3.53 | -0.14 | -5.82 – -1.24 | -0.23 – -0.05 | **0.003** |
| Sex | 0.09 | 0.00 | -3.12 – 3.30 | -0.12 – 0.13 | 0.954 |
| Testosterone × Cortisol | -0.02 | -0.00 | -2.15 – 2.12 | -0.09 – 0.08 | 0.987 |
| Testosterone × Sex | 0.62 | 0.02 | -2.66 – 3.89 | -0.11 – 0.15 | 0.712 |
| Cortisol × Sex | 4.52 | 0.18 | 1.30 – 7.75 | 0.05 – 0.31 | **0.006** |
| Testosterone × Cortisol × Sex | -0.48 | -0.02 | -3.43 – 2.47 | -0.14 – 0.10 | 0.748 |
| Observations | 990 | | | | |
| R^2^ / R^2^ adjusted | 0.011 / 0.004 | | | | |
| F(7,982) = 1.55, p = 0.15 | | | | | |

**Table S10.** Results from logistic regression analyses of “amount offered” in the ultimatum game including covariates.

|  | **Amount offered** | | | | |
| --- | --- | --- | --- | --- | --- |
| **Step 1** | *Estimates* | *std. Beta* | *CI* | *standardized CI* | *p* |
| (Intercept) | 73.74 | 0.11 | 62.87 – 84.62 | -0.04 – 0.27 | **<0.001** |
| Testosterone | 0.70 | 0.03 | -0.98 – 2.38 | -0.04 – 0.09 | 0.414 |
| Cortisol | -1.41 | -0.06 | -3.08 – 0.26 | -0.12 – 0.01 | 0.099 |
| Sex | 0.03 | 0.00 | -3.75 – 3.80 | -0.15 – 0.15 | 0.988 |
| CollectionWeek | 0.16 | 0.04 | -0.12 – 0.44 | -0.03 – 0.10 | 0.259 |
| Hair type | -4.99 | -0.20 | -11.11 – 1.12 | -0.44 – 0.04 | 0.109 |
| Contraceptives | -1.51 | -0.06 | -6.36 – 3.34 | -0.25 – 0.13 | 0.541 |
| BMI | -0.00 | -0.00 | -0.39 – 0.38 | -0.07 – 0.06 | 0.988 |
| Hair color | -2.97 | -0.12 | -6.88 – 0.95 | -0.27 – 0.04 | 0.137 |
| Opioids | 0.31 | 0.01 | -1.28 – 1.91 | -0.05 – 0.08 | 0.700 |
| Cannabis | 0.68 | 0.04 | -0.43 – 1.78 | -0.03 – 0.11 | 0.229 |
| Stimulants | 1.78 | 0.02 | -5.23 – 8.79 | -0.06 – 0.09 | 0.618 |
| MDMA | -0.38 | -0.02 | -1.62 – 0.87 | -0.10 – 0.05 | 0.553 |
| Observations | 985 | | | | |
| R^2^ / R^2^ adjusted | 0.010 / -0.002 | | | | |
| F(12,972) = 0.83, p = 0.62 | | | | | |

| **Step 2** | *Estimates* | *std. Beta* | *CI* | *standardized CI* | *p* |
| --- | --- | --- | --- | --- | --- |
| (Intercept) | 73.75 | 0.12 | 62.87 – 84.63 | -0.04 – 0.27 | **<0.001** |
| Testosterone | 0.78 | 0.03 | -0.94 – 2.50 | -0.04 – 0.10 | 0.372 |
| Cortisol | -1.42 | -0.06 | -3.10 – 0.25 | -0.12 – 0.01 | 0.096 |
| Sex | 0.05 | 0.00 | -3.73 – 3.83 | -0.15 – 0.15 | 0.980 |
| CollectionWeek | 0.16 | 0.04 | -0.12 – 0.44 | -0.03 – 0.10 | 0.257 |
| Hair type | -4.92 | -0.19 | -11.04 – 1.21 | -0.43 – 0.05 | 0.115 |
| Contraceptives | -1.50 | -0.06 | -6.35 – 3.36 | -0.25 – 0.13 | 0.546 |
| BMI | 0.00 | 0.00 | -0.39 – 0.39 | -0.06 – 0.06 | 1.000 |
| Hair color | -3.02 | -0.12 | -6.95 – 0.90 | -0.27 – 0.04 | 0.131 |
| Opioids | 0.28 | 0.01 | -1.31 – 1.88 | -0.05 – 0.08 | 0.727 |
| Cannabis | 0.68 | 0.04 | -0.42 – 1.79 | -0.03 – 0.11 | 0.225 |
| Stimulants | 1.78 | 0.02 | -5.23 – 8.79 | -0.06 – 0.09 | 0.619 |
| MDMA | -0.39 | -0.02 | -1.64 – 0.86 | -0.10 – 0.05 | 0.543 |
| Testosterone × Cortisol | -0.35 | -0.01 | -1.84 – 1.15 | -0.07 – 0.05 | 0.649 |
| Observations | 985 | | | | |
| R^2^ / R^2^ adjusted | 0.010 / -0.003 | | | | |
| F(13,971) = 0.78, p = 0.68 | | | | | |

# Ultimatum game: minimum acceptable offer

**Table S11.** Results from logistic regression analyses of “minimum acceptable offer” in the ultimatum game including the interaction term with sex.

|  | **Minimum acceptable offer** | | | | |
| --- | --- | --- | --- | --- | --- |
| **Step 1** | *Estimates* | *std. Beta* | *CI* | *standardized CI* | *p* |
| (Intercept) | 55.81 | -0.05 | 53.43 – 58.20 | -0.14 – 0.03 | **<0.001** |
| Testosterone | 0.43 | 0.02 | -1.99 – 2.84 | -0.07 – 0.10 | 0.728 |
| Cortisol | 1.57 | 0.06 | -0.88 – 4.02 | -0.03 – 0.15 | 0.210 |
| Sex | 2.87 | 0.11 | -0.51 – 6.26 | -0.02 – 0.23 | 0.096 |
| Testosterone × Sex | -1.37 | -0.05 | -4.81 – 2.08 | -0.18 – 0.08 | 0.437 |
| Cortisol × Sex | -0.38 | -0.01 | -3.84 – 3.07 | -0.14 – 0.11 | 0.828 |
| Observations | 1001 | | | | |
| R^2^ / R^2^ adjusted | 0.006 / 0.001 | | | | |
| F(5,995) = 1.11, p = 0.35 | | | | | |

| **Step 2** | *Estimates* | *std. Beta* | *CI* | *standardized CI* | *p* |
| --- | --- | --- | --- | --- | --- |
| (Intercept) | 55.85 | -0.05 | 53.44 – 58.27 | -0.14 – 0.04 | **<0.001** |
| Testosterone | 0.49 | 0.02 | -1.99 – 2.96 | -0.07 – 0.11 | 0.700 |
| Cortisol | 1.56 | 0.06 | -0.89 – 4.01 | -0.03 – 0.15 | 0.212 |
| Sex | 2.68 | 0.10 | -0.76 – 6.12 | -0.03 – 0.22 | 0.127 |
| Testosterone × Cortisol | -0.25 | -0.01 | -2.54 – 2.05 | -0.09 – 0.08 | 0.834 |
| Testosterone × Sex | -1.57 | -0.06 | -5.09 – 1.95 | -0.19 – 0.07 | 0.381 |
| Cortisol × Sex | -0.32 | -0.01 | -3.79 – 3.14 | -0.14 – 0.12 | 0.854 |
| Testosterone × Cortisol × Sex | 0.95 | 0.03 | -2.22 – 4.12 | -0.08 – 0.15 | 0.555 |
| Observations | 1001 | | | | |
| R^2^ / R^2^ adjusted | 0.006 / -0.001 | | | | |
| F(7,993) = 0.85, p = 0.54 | | | | | |

**Table S12.** Results from logistic regression analyses of “minimum acceptable offer” in the ultimatum game including covariates.

|  | **Minimum acceptable offer** | | | | |
| --- | --- | --- | --- | --- | --- |
| **Step 1** | *Estimates* | *std. Beta* | *CI* | *standardized CI* | *p* |
| (Intercept) | 41.96 | -0.10 | 30.43 – 53.48 | -0.25 – 0.06 | **<0.001** |
| Testosterone | -0.60 | -0.02 | -2.38 – 1.18 | -0.09 – 0.04 | 0.508 |
| Cortisol | 0.98 | 0.04 | -0.79 – 2.76 | -0.03 – 0.10 | 0.277 |
| Sex | 1.18 | 0.04 | -2.82 – 5.17 | -0.10 – 0.19 | 0.564 |
| CollectionWeek | -0.13 | -0.03 | -0.43 – 0.17 | -0.09 – 0.04 | 0.404 |
| Hair type | 2.44 | 0.09 | -4.02 – 8.90 | -0.15 – 0.33 | 0.459 |
| Contraceptives | 0.96 | 0.04 | -4.18 – 6.09 | -0.15 – 0.22 | 0.715 |
| BMI | 0.58 | 0.09 | 0.17 – 0.99 | 0.03 – 0.15 | **0.005** |
| Hair color | 2.19 | 0.08 | -1.97 – 6.34 | -0.07 – 0.23 | 0.302 |
| Opioids | 0.63 | 0.02 | -1.04 – 2.30 | -0.04 – 0.09 | 0.459 |
| Cannabis | 1.21 | 0.07 | 0.04 – 2.38 | 0.00 – 0.14 | **0.042** |
| Stimulants | -3.98 | -0.04 | -11.43 – 3.48 | -0.11 – 0.03 | 0.296 |
| MDMA | 0.22 | 0.01 | -1.09 – 1.54 | -0.06 – 0.09 | 0.740 |
| Observations | 996 | | | | |
| R^2^ / R^2^ adjusted | 0.021 / 0.009 | | | | |
| F(12,983) = 1.72, p = 0.06 | | | | | |

| **Step 2** | *Estimates* | *std. Beta* | *CI* | *standardized CI* | *p* |
| --- | --- | --- | --- | --- | --- |
| (Intercept) | 41.95 | -0.10 | 30.42 – 53.48 | -0.25 – 0.06 | **<0.001** |
| Testosterone | -0.67 | -0.02 | -2.49 – 1.16 | -0.09 – 0.04 | 0.474 |
| Cortisol | 1.00 | 0.04 | -0.78 – 2.77 | -0.03 – 0.10 | 0.272 |
| Sex | 1.16 | 0.04 | -2.84 – 5.16 | -0.10 – 0.19 | 0.570 |
| CollectionWeek | -0.13 | -0.03 | -0.43 – 0.17 | -0.09 – 0.04 | 0.401 |
| Hair type | 2.39 | 0.09 | -4.09 – 8.86 | -0.15 – 0.33 | 0.470 |
| Contraceptives | 0.94 | 0.03 | -4.20 – 6.08 | -0.15 – 0.22 | 0.719 |
| BMI | 0.58 | 0.09 | 0.17 – 0.99 | 0.03 – 0.15 | **0.006** |
| Hair color | 2.23 | 0.08 | -1.93 – 6.40 | -0.07 – 0.24 | 0.293 |
| Opioids | 0.65 | 0.03 | -1.02 – 2.33 | -0.04 – 0.09 | 0.444 |
| Cannabis | 1.21 | 0.07 | 0.04 – 2.38 | 0.00 – 0.14 | **0.043** |
| Stimulants | -3.97 | -0.04 | -11.43 – 3.49 | -0.11 – 0.03 | 0.296 |
| MDMA | 0.23 | 0.01 | -1.09 – 1.54 | -0.06 – 0.09 | 0.734 |
| Testosterone × Cortisol | 0.27 | 0.01 | -1.32 – 1.85 | -0.05 – 0.07 | 0.738 |
| Observations | 996 | | | | |
| R^2^ / R^2^ adjusted | 0.021 / 0.008 | | | | |
| F(13,982) = 1.60, p = 0.08 | | | | | |

# Public goods game: amount contributed

**Table S13.** Results from logistic regression analyses of “amount contributed” in the public goods game including the interaction term with sex.

|  | **Amount contributed** | | | | |
| --- | --- | --- | --- | --- | --- |
| **Step 1** | *Estimates* | *std. Beta* | *CI* | *standardized CI* | *p* |
| (Intercept) | 33.32 | -0.00 | 31.87 – 34.77 | -0.09 – 0.08 | **<0.001** |
| Testosterone | -0.13 | -0.01 | -1.60 – 1.34 | -0.10 – 0.08 | 0.863 |
| Cortisol | -0.45 | -0.03 | -1.94 – 1.03 | -0.12 – 0.06 | 0.549 |
| Sex | 0.15 | 0.01 | -1.90 – 2.20 | -0.12 – 0.13 | 0.888 |
| Testosterone × Sex | -0.59 | -0.04 | -2.69 – 1.51 | -0.16 – 0.09 | 0.581 |
| Cortisol × Sex | -0.10 | -0.01 | -2.19 – 1.99 | -0.13 – 0.12 | 0.926 |
| Observations | 987 | | | | |
| R^2^ / R^2^ adjusted | 0.002 / -0.003 | | | | |
| F(5,981) = 0.46, p = 0.80 | | | | | |

| **Step 2** | *Estimates* | *std. Beta* | *CI* | *standardized CI* | *p* |
| --- | --- | --- | --- | --- | --- |
| (Intercept) | 33.29 | -0.01 | 31.83 – 34.75 | -0.10 – 0.08 | **<0.001** |
| Testosterone | -0.18 | -0.01 | -1.68 – 1.33 | -0.10 – 0.08 | 0.819 |
| Cortisol | -0.45 | -0.03 | -1.93 – 1.04 | -0.12 – 0.06 | 0.556 |
| Sex | 0.06 | 0.00 | -2.02 – 2.15 | -0.12 – 0.13 | 0.952 |
| Testosterone × Cortisol | 0.23 | 0.01 | -1.19 – 1.65 | -0.07 – 0.10 | 0.749 |
| Testosterone × Sex | -0.65 | -0.04 | -2.78 – 1.49 | -0.17 – 0.09 | 0.551 |
| Cortisol × Sex | -0.07 | -0.00 | -2.16 – 2.03 | -0.13 – 0.12 | 0.949 |
| Testosterone × Cortisol × Sex | 0.28 | 0.02 | -1.65 – 2.22 | -0.10 – 0.13 | 0.774 |
| Observations | 987 | | | | |
| R^2^ / R^2^ adjusted | 0.003 / -0.004 | | | | |
| F(7,979) = 0.43, p = 0.88 | | | | | |

**Table S14.** Results from logistic regression analyses of “amount contributed” in the public goods game including covariates.

|  | **Amount contributed** | | | | |
| --- | --- | --- | --- | --- | --- |
| **Step 1** | *Estimates* | *std. Beta* | *CI* | *standardized CI* | *p* |
| (Intercept) | 37.79 | -0.01 | 30.76 – 44.82 | -0.16 – 0.14 | **<0.001** |
| Testosterone | -0.33 | -0.02 | -1.42 – 0.76 | -0.09 – 0.05 | 0.549 |
| Cortisol | -0.23 | -0.01 | -1.31 – 0.84 | -0.08 – 0.05 | 0.671 |
| Sex | 0.58 | 0.04 | -1.86 – 3.01 | -0.11 – 0.18 | 0.642 |
| CollectionWeek | -0.09 | -0.03 | -0.27 – 0.09 | -0.10 – 0.03 | 0.336 |
| Hair type | 2.57 | 0.16 | -1.37 – 6.52 | -0.08 – 0.40 | 0.201 |
| Contraceptives | 1.09 | 0.07 | -2.03 – 4.22 | -0.12 – 0.26 | 0.493 |
| BMI | -0.11 | -0.03 | -0.36 – 0.14 | -0.09 – 0.04 | 0.398 |
| Hair color | -0.68 | -0.04 | -3.20 – 1.84 | -0.20 – 0.11 | 0.597 |
| Opioids | 0.01 | 0.00 | -1.02 – 1.03 | -0.06 – 0.07 | 0.992 |
| Cannabis | 0.08 | 0.01 | -0.63 – 0.79 | -0.06 – 0.08 | 0.819 |
| Stimulants | -0.55 | -0.01 | -5.38 – 4.28 | -0.08 – 0.07 | 0.823 |
| MDMA | -0.60 | -0.06 | -1.40 – 0.21 | -0.14 – 0.02 | 0.145 |
| Observations | 982 | | | | |
| R^2^ / R^2^ adjusted | 0.009 / -0.003 | | | | |
| F(12,969) = 0.74, p = 0.72 | | | | | |

| **Step 2** | *Estimates* | *std. Beta* | *CI* | *standardized CI* | *p* |
| --- | --- | --- | --- | --- | --- |
| (Intercept) | 37.78 | -0.01 | 30.75 – 44.82 | -0.17 – 0.14 | **<0.001** |
| Testosterone | -0.40 | -0.02 | -1.51 – 0.71 | -0.09 – 0.04 | 0.477 |
| Cortisol | -0.22 | -0.01 | -1.30 – 0.86 | -0.08 – 0.05 | 0.693 |
| Sex | 0.55 | 0.03 | -1.88 – 2.99 | -0.12 – 0.18 | 0.655 |
| CollectionWeek | -0.09 | -0.03 | -0.27 – 0.09 | -0.10 – 0.03 | 0.331 |
| Hair type | 2.51 | 0.15 | -1.45 – 6.46 | -0.09 – 0.40 | 0.214 |
| Contraceptives | 1.08 | 0.07 | -2.05 – 4.20 | -0.13 – 0.26 | 0.500 |
| BMI | -0.11 | -0.03 | -0.36 – 0.14 | -0.09 – 0.04 | 0.386 |
| Hair color | -0.63 | -0.04 | -3.16 – 1.90 | -0.19 – 0.12 | 0.626 |
| Opioids | 0.03 | 0.00 | -1.00 – 1.06 | -0.06 – 0.07 | 0.954 |
| Cannabis | 0.08 | 0.01 | -0.63 – 0.79 | -0.06 – 0.08 | 0.832 |
| Stimulants | -0.56 | -0.01 | -5.39 – 4.27 | -0.08 – 0.07 | 0.820 |
| MDMA | -0.59 | -0.06 | -1.39 – 0.22 | -0.13 – 0.02 | 0.152 |
| Testosterone × Cortisol | 0.32 | 0.02 | -0.65 – 1.29 | -0.04 – 0.08 | 0.521 |
| Observations | 982 | | | | |
| R^2^ / R^2^ adjusted | 0.009 / -0.004 | | | | |
| F(13,968) = 0.71, p = 0.75 | | | | | |

# Self-reported aggression

**Table S15.** Results from logistic regression analyses of “self-reported aggression” (SBQ) including the interaction term with sex.

|  | **Aggression** | | | | |
| --- | --- | --- | --- | --- | --- |
| **Step 1** | *Estimates* | *std. Beta* | *CI* | *standardized CI* | *p* |
| (Intercept) | 1.39 | -0.09 | 1.36 – 1.42 | -0.18 – -0.01 | **<0.001** |
| Testosterone | 0.04 | 0.12 | 0.01 – 0.07 | 0.03 – 0.20 | **0.009** |
| Cortisol | 0.01 | 0.03 | -0.02 – 0.04 | -0.06 – 0.12 | 0.506 |
| Sex | 0.07 | 0.19 | 0.02 – 0.11 | 0.06 – 0.31 | **0.003** |
| Testosterone × Sex | -0.03 | -0.07 | -0.07 – 0.02 | -0.20 – 0.05 | 0.258 |
| Cortisol × Sex | 0.00 | 0.01 | -0.04 – 0.05 | -0.12 – 0.13 | 0.903 |
| Observations | 1001 | | | | |
| R^2^ / R^2^ adjusted | 0.018 / 0.013 | | | | |
| F(5,995) = 3.71, p < 0.001 | | | | | |

| **Step 2** | *Estimates* | *std. Beta* | *CI* | *standardized CI* | *p* |
| --- | --- | --- | --- | --- | --- |
| (Intercept) | 1.39 | -0.09 | 1.36 – 1.42 | -0.18 – -0.00 | **<0.001** |
| Testosterone | 0.04 | 0.12 | 0.01 – 0.07 | 0.03 – 0.21 | **0.010** |
| Cortisol | 0.01 | 0.03 | -0.02 – 0.04 | -0.06 – 0.12 | 0.509 |
| Sex | 0.06 | 0.16 | 0.01 – 0.10 | 0.04 – 0.29 | **0.010** |
| Testosterone × Cortisol | -0.00 | -0.01 | -0.03 – 0.03 | -0.09 – 0.07 | 0.838 |
| Testosterone × Sex | -0.03 | -0.09 | -0.08 – 0.01 | -0.22 – 0.03 | 0.148 |
| Cortisol × Sex | 0.01 | 0.01 | -0.04 – 0.05 | -0.11 – 0.14 | 0.815 |
| Testosterone × Cortisol × Sex | 0.04 | 0.10 | -0.00 – 0.08 | -0.01 – 0.22 | 0.081 |
| Observations | 1001 | | | | |
| R^2^ / R^2^ adjusted | 0.024 / 0.017 | | | | |
| F(7,993) = 3.44, p < 0.001 | | | | | |

**Table S16.** Results from logistic regression analyses of “self-reported aggression” (SBQ) including covariates.

|  | **Aggression** | | | | |
| --- | --- | --- | --- | --- | --- |
| **Step 1** | *Estimates* | *std. Beta* | *CI* | *standardized CI* | *p* |
| (Intercept) | 1.28 | -0.14 | 1.14 – 1.43 | -0.29 – 0.01 | **<0.001** |
| Testosterone | 0.02 | 0.06 | -0.00 – 0.04 | -0.01 – 0.12 | 0.071 |
| Cortisol | 0.00 | 0.00 | -0.02 – 0.02 | -0.06 – 0.06 | 0.974 |
| Sex | 0.02 | 0.05 | -0.03 – 0.07 | -0.09 – 0.20 | 0.462 |
| CollectionWeek | 0.00 | 0.02 | -0.00 – 0.01 | -0.04 – 0.08 | 0.479 |
| Hair type | 0.08 | 0.22 | -0.00 – 0.16 | -0.01 – 0.45 | 0.065 |
| Contraceptives | 0.01 | 0.03 | -0.05 – 0.07 | -0.16 – 0.21 | 0.785 |
| BMI | 0.00 | 0.02 | -0.00 – 0.01 | -0.04 – 0.09 | 0.442 |
| Hair color | 0.04 | 0.12 | -0.01 – 0.09 | -0.03 – 0.27 | 0.104 |
| Opioids | 0.04 | 0.11 | 0.02 – 0.06 | 0.05 – 0.17 | **<0.001** |
| Cannabis | 0.02 | 0.11 | 0.01 – 0.04 | 0.04 – 0.17 | **0.002** |
| Stimulants | 0.16 | 0.12 | 0.06 – 0.25 | 0.05 – 0.19 | **0.001** |
| MDMA | 0.00 | 0.01 | -0.01 – 0.02 | -0.07 – 0.08 | 0.824 |
| Observations | 996 | | | | |
| R^2^ / R^2^ adjusted | 0.084 / 0.073 | | | | |
| F(12,983) = 7.49, p < 0.001 | | | | | |

| **Step 2** | *Estimates* | *std. Beta* | *CI* | *standardized CI* | *p* |
| --- | --- | --- | --- | --- | --- |
| (Intercept) | 1.28 | -0.16 | 1.14 – 1.43 | -0.31 – -0.01 | **<0.001** |
| Testosterone | 0.02 | 0.04 | -0.01 – 0.04 | -0.02 – 0.11 | 0.179 |
| Cortisol | 0.00 | 0.00 | -0.02 – 0.02 | -0.06 – 0.07 | 0.911 |
| Sex | 0.02 | 0.05 | -0.03 – 0.07 | -0.09 – 0.19 | 0.494 |
| CollectionWeek | 0.00 | 0.02 | -0.00 – 0.00 | -0.04 – 0.08 | 0.494 |
| Hair type | 0.07 | 0.21 | -0.01 – 0.15 | -0.02 – 0.43 | 0.080 |
| Contraceptives | 0.01 | 0.02 | -0.06 – 0.07 | -0.16 – 0.21 | 0.809 |
| BMI | 0.00 | 0.02 | -0.00 – 0.01 | -0.04 – 0.08 | 0.482 |
| Hair color | 0.05 | 0.13 | -0.01 – 0.10 | -0.02 – 0.28 | 0.080 |
| Opioids | 0.04 | 0.12 | 0.02 – 0.06 | 0.05 – 0.18 | **<0.001** |
| Cannabis | 0.02 | 0.10 | 0.01 – 0.04 | 0.04 – 0.17 | **0.002** |
| Stimulants | 0.16 | 0.12 | 0.07 – 0.25 | 0.05 – 0.19 | **0.001** |
| MDMA | 0.00 | 0.01 | -0.01 – 0.02 | -0.06 – 0.09 | 0.785 |
| Testosterone × Cortisol | 0.02 | 0.06 | 0.00 – 0.04 | 0.00 – 0.11 | **0.045** |
| Observations | 996 | | | | |
| R^2^ / R^2^ adjusted | 0.087 / 0.075 | | | | |
| F(13,982) = 7.24, p < 0.001 | | | | | |

# Self-reported prosociality

**Table S17.** Results from logistic regression analyses of “self-reported prosociality” (SBQ) including the interaction term with sex.

|  | **Prosociality** | | | | |
| --- | --- | --- | --- | --- | --- |
| **Step 1** | *Estimates* | *std. Beta* | *CI* | *standardized CI* | *p* |
| (Intercept) | 3.94 | 0.22 | 3.90 – 3.99 | 0.13 – 0.30 | **<0.001** |
| Testosterone | 0.02 | 0.04 | -0.02 – 0.07 | -0.04 – 0.13 | 0.338 |
| Cortisol | 0.04 | 0.07 | -0.01 – 0.09 | -0.02 – 0.15 | 0.134 |
| Sex | -0.25 | -0.44 | -0.31 – -0.18 | -0.57 – -0.32 | **<0.001** |
| Testosterone × Sex | 0.00 | 0.00 | -0.07 – 0.07 | -0.12 – 0.13 | 0.972 |
| Cortisol × Sex | -0.06 | -0.11 | -0.13 – 0.01 | -0.23 – 0.01 | 0.082 |
| Observations | 1001 | | | | |
| R^2^ / R^2^ adjusted | 0.055 / 0.050 | | | | |
| F(5,995) = 11.54, p < 0.001 | | | | | |

| **Step 2** | *Estimates* | *std. Beta* | *CI* | *standardized CI* | *p* |
| --- | --- | --- | --- | --- | --- |
| (Intercept) | 3.95 | 0.22 | 3.90 – 3.99 | 0.14 – 0.31 | **<0.001** |
| Testosterone | 0.03 | 0.05 | -0.02 – 0.08 | -0.04 – 0.14 | 0.261 |
| Cortisol | 0.04 | 0.07 | -0.01 – 0.09 | -0.02 – 0.15 | 0.139 |
| Sex | -0.24 | -0.44 | -0.31 – -0.17 | -0.56 – -0.31 | **<0.001** |
| Testosterone × Cortisol | -0.02 | -0.04 | -0.07 – 0.03 | -0.12 – 0.05 | 0.394 |
| Testosterone × Sex | 0.00 | 0.01 | -0.07 – 0.07 | -0.12 – 0.13 | 0.923 |
| Cortisol × Sex | -0.06 | -0.11 | -0.13 – 0.01 | -0.24 – 0.01 | 0.073 |
| Testosterone × Cortisol × Sex | -0.01 | -0.02 | -0.08 – 0.05 | -0.14 – 0.09 | 0.686 |
| Observations | 1001 | | | | |
| R^2^ / R^2^ adjusted | 0.058 / 0.051 | | | | |
| F(7,993) = 8.67, p < 0.001 | | | | | |

**Table S18.** Results from logistic regression analyses of “self-reported prosociality” (SBQ) including covariates.

|  | **Prosociality** | | | | |
| --- | --- | --- | --- | --- | --- |
| **Step 1** | *Estimates* | *std. Beta* | *CI* | *standardized CI* | *p* |
| (Intercept) | 3.92 | 0.21 | 3.69 – 4.15 | 0.06 – 0.36 | **<0.001** |
| Testosterone | 0.03 | 0.05 | -0.01 – 0.06 | -0.02 – 0.11 | 0.148 |
| Cortisol | 0.01 | 0.01 | -0.03 – 0.04 | -0.05 – 0.08 | 0.704 |
| Sex | -0.21 | -0.39 | -0.29 – -0.13 | -0.53 – -0.24 | **<0.001** |
| CollectionWeek | 0.00 | 0.01 | -0.00 – 0.01 | -0.05 – 0.08 | 0.686 |
| Hair type | -0.08 | -0.14 | -0.21 – 0.05 | -0.38 – 0.09 | 0.223 |
| Contraceptives | 0.07 | 0.12 | -0.04 – 0.17 | -0.06 – 0.31 | 0.202 |
| BMI | -0.00 | -0.01 | -0.01 – 0.01 | -0.07 – 0.05 | 0.764 |
| Hair color | -0.02 | -0.03 | -0.10 – 0.06 | -0.18 – 0.12 | 0.651 |
| Opioids | 0.03 | 0.05 | -0.01 – 0.06 | -0.01 – 0.12 | 0.098 |
| Cannabis | 0.01 | 0.02 | -0.02 – 0.03 | -0.05 – 0.09 | 0.507 |
| Stimulants | -0.06 | -0.03 | -0.21 – 0.09 | -0.10 – 0.04 | 0.423 |
| MDMA | 0.00 | 0.01 | -0.02 – 0.03 | -0.07 – 0.08 | 0.878 |
| Observations | 996 | | | | |
| R^2^ / R^2^ adjusted | 0.059 / 0.047 | | | | |
| F(12,983) = 5.09, p < 0.001 | | | | | |

| **Step 2** | *Estimates* | *std. Beta* | *CI* | *standardized CI* | *p* |
| --- | --- | --- | --- | --- | --- |
| (Intercept) | 3.92 | 0.22 | 3.69 – 4.15 | 0.07 – 0.37 | **<0.001** |
| Testosterone | 0.03 | 0.06 | -0.00 – 0.07 | -0.01 – 0.12 | 0.084 |
| Cortisol | 0.01 | 0.01 | -0.03 – 0.04 | -0.05 – 0.07 | 0.748 |
| Sex | -0.21 | -0.38 | -0.29 – -0.13 | -0.53 – -0.24 | **<0.001** |
| CollectionWeek | 0.00 | 0.01 | -0.00 – 0.01 | -0.05 – 0.08 | 0.672 |
| Hair type | -0.08 | -0.14 | -0.20 – 0.05 | -0.37 – 0.10 | 0.253 |
| Contraceptives | 0.07 | 0.12 | -0.03 – 0.17 | -0.06 – 0.31 | 0.194 |
| BMI | -0.00 | -0.01 | -0.01 – 0.01 | -0.07 – 0.05 | 0.802 |
| Hair color | -0.02 | -0.04 | -0.11 – 0.06 | -0.19 – 0.11 | 0.585 |
| Opioids | 0.03 | 0.05 | -0.01 – 0.06 | -0.01 – 0.11 | 0.124 |
| Cannabis | 0.01 | 0.02 | -0.02 – 0.03 | -0.04 – 0.09 | 0.482 |
| Stimulants | -0.06 | -0.03 | -0.21 – 0.09 | -0.10 – 0.04 | 0.420 |
| MDMA | 0.00 | 0.00 | -0.02 – 0.03 | -0.07 – 0.08 | 0.907 |
| Testosterone × Cortisol | -0.02 | -0.04 | -0.06 – 0.01 | -0.10 – 0.01 | 0.139 |
| Observations | 996 | | | | |
| R^2^ / R^2^ adjusted | 0.061 / 0.048 | | | | |
| F(13,982) = 4.88, p < 0.001 | | | | | |
